# Factors affecting the number and type of student research products for chemistry and physics students at primarily undergraduate institutions: A case study

**DOI:** 10.1371/journal.pone.0196338

**Published:** 2018-04-26

**Authors:** Birgit Mellis, Patricia Soto, Chrystal D. Bruce, Graciela Lacueva, Anne M. Wilson, Rasitha Jayasekare

**Affiliations:** 1 Department of Chemistry and Physics, University of St Thomas, Houston, Texas, United States of America; 2 Department of Physics, Creighton University, Omaha, Nebraska, United States of America; 3 Department of Chemistry, John Carroll University, University Heights, Ohio, United States of America; 4 Department of Physics, John Carroll University, University Heights, Ohio, United States of America; 5 Department of Chemistry, Butler University, Indianapolis, Indiana, United States of America; 6 Department of Mathematics, Statistics and Actuarial Science, Butler University, Indianapolis, Indiana, United States of America; Universite de Montreal, CANADA

## Abstract

For undergraduate students, involvement in authentic research represents scholarship that is consistent with disciplinary quality standards and provides an integrative learning experience. In conjunction with performing research, the communication of the results via presentations or publications is a measure of the level of scientific engagement. The empirical study presented here uses generalized linear mixed models with hierarchical bootstrapping to examine the factors that impact the means of dissemination of undergraduate research results. Focusing on the research experiences in physics and chemistry of undergraduates at four Primarily Undergraduate Institutions (PUIs) from 2004–2013, statistical analysis indicates that the gender of the student does not impact the number and type of research products. However, in chemistry, the rank of the faculty advisor and the venue of the presentation do impact the number of research products by undergraduate student, whereas in physics, gender match between student and advisor has an effect on the number of undergraduate research products. This study provides a baseline for future studies of discipline-based bibliometrics and factors that affect the number of research products of undergraduate students.

## Introduction

The importance of high impact educational practices to strengthen students' undergraduate experiences has been validated by longitudinal studies, and practices like mentored research are an integral part of the future of undergraduate education [1]. In particular, programs for engaging undergraduate students in STEM (Science, Technology, Engineering, and Mathematics) disciplines, which appear to be particularly important for retaining female students, abound at academic institutions across the country [2–4]. The quantitative and qualitative value of undergraduate research as a high impact educational practice, regardless of the type of institution (liberal arts college, PUI, research-intensive, etc.), has been recently and extensively examined in the literature [5–15]. Ranging from retention programs, career promotion programs, research apprenticeships, and research-based learning [5], undergraduate research experiences are an important component in preparing scientists in the STEM fields. According to these studies, undergraduate research deepens the undergraduate experience, is a key indicator for student success, is a measure of a quality program, and is an excellent training ground for early scientists.

As a training platform for future scientists, research experiences can contribute to growth in all areas identified as needs of the STEM workforce for the coming century: oral, written, and computer communication; self-motivation; passion and enthusiasm; technical and intellectual abilities; problem solving and critical thinking abilities; and the pursuit of further education or training [15–22]. While working on a research project can strengthen these much needed skills, undergraduate research experiences are not without some identified weaknesses, especially in the dissemination of research results [5]. Students should be given the opportunity to present their results to benefit most from the research experience. In one study, undergraduates reported that the single greatest benefit of attending conferences was having the opportunity to present their research, and they reported their experience as positive or “life changing” [23]. However, in a study conducted by Seymoure et al, only a few students presented at a regional or national scientific meeting (7% presented a poster, 9% presented a paper), expected to have their results incorporated into a professional publication (7% had done so, 21% expected to do so), or utilized their undergraduate research as the basis for an undergraduate thesis (7%) [5]. A study of the perceptions of undergraduates who presented at a national meeting showed this was a positive professional development experience [23], but the results were limited to undergraduates who presented at two national meetings. A more detailed analysis that includes all kinds of undergraduate research products is still needed.

Considering the positive impact on students of disseminating their research results, the study reported here, conducted at four Primarily Undergraduate Institutions (PUIs) over a ten-year period from 2004–2013, examines what factors impact the number of students who present their work at local, regional, or national venues in poster or oral format, or have contributed to a peer-reviewed publication. The factors that were considered to possibly impact whether or not a student presented their results include discipline (chemistry or physics), faculty rank (assistant, associate, full professor), venue location (local, regional, national), presentation type (poster, paper, oral), self-identified gender of faculty member, and self-identified gender of research student. Gender was specifically included as a potential factor with the goal that examination of any gender difference at the undergraduate research experience in our PUIs might shed a light on gender differences seen at higher levels in chemistry and physics or other STEM disciplines. Pezzoni et al published a study showing some gender effects in the publication output of graduate students at a research-intensive university [24]. In particular, they find that gender pairing matters: male students working with female advisors publish 10.0% more than male students working with male advisors; women students working with male advisors publish 8.5% less. An equivalent analysis for publication trends of undergraduate students is lacking, as well as metrics to assess the impact of undergraduate research [25]. The focus of this study was on PUIs, which are important contributors in STEM workforce education [3]. Students from private non-doctoral institutions like those in this study tend to have a high rate of undergraduate persistence in STEM fields, obtain their STEM degrees in four years or less, and account for about 25% of PhD candidates in the physical sciences [3]. There are a limited number of studies that focus on the research experiences of STEM students at PUIs. Therefore the authors, all faculty at PUIs, collected and analyzed data from those institutions.

While not news, there have been continued reports of “leaky pipelines,” “gender filters,” and related challenges [26–32]. Hazari and Potvin [33] point out the need for a deeper understanding of the factors that influence the decision of students to persevere in a science (physics) career. Of the students who took the AP tests in 2005, which may be an indicator of interest in these areas of study, women comprised 46.5% of those taking the chemistry test and 35.3% of those taking the physics test [34]. Nine years later in 2014, the percentage of PhDs awarded to women in chemistry was 39.4%, and the percentage of PhDs awarded to women in physics was 18.7% [35]. While this data does not indicate why or where the percentage decreased, it is clear that there is a downward trend. Finding ways to mitigate this attrition is important as the percentage of women interested in chemistry and physics is increasing; in 2016, 49.6% of chemistry AP (Advanced Placement) test takers were female, and 37.3% physics AP test takers were women [36]. Undergraduate research has been seen as one possible tool to improve retention of female STEM students [5, 8, 12, 17]. A recent comparison of thesis production and peer-reviewed publication between a few institutions has been reported [37], and this single study suggests that women are, for the most part, as engaged as their male counterparts by these measures.

The impact of gender bias in STEM disciplines has been gaining broader public awareness [38]. Women who are successful in STEM disciplines seem to be as successful as their male counterparts [39, 40], but there is clearly still vigorous debate on this issue [41]. A longitudinal study has indicated a gender difference in self-confidence of STEM professionals [32, 42]. Other studies have shown that gender differences are measurable in academic literature authorship across a wide range of disciplines [43–45]. Despite awareness initiatives, a recent study found that men failed to acknowledge the existence of gender differences or bias, despite evidence indicating that such differences or bias exists [46]. Several groups have found that diverse role models have a positive impact on women in STEM disciplines [47–49]. Because faculty research mentors are role models, this study examines the impact of faculty rank and faculty-student gender match on research productivity of both male and female undergraduate research students.

## Motivation

The two fundamental issues motivating this inquiry are that undergraduate research experiences are of great value for students’ education and career development and that the dissemination of research is an integral part of the research process. Offering students opportunities for the dissemination of their research in a variety of venues completes the research cycle, providing students a deeper understanding of the research process as well as helping the development of their communication skills [50]. As mentioned earlier, a number of studies have shown that students benefit from engaging in undergraduate research. However, a detailed analysis of the factors influencing the dissemination of their research results is lacking. To account for the paucity of data in this area, this study presents an evaluation of ten years of physics and chemistry undergraduate research at four PUIs investigating the factors that influence the dissemination of undergraduate research in the form of posters, oral presentations, and papers. Such factors include faculty rank, faculty discipline, and gender of both faculty and student researchers.

## Methods

### Participants

The authors of this study are part of an NSF (National Science Foundation) ADVANCE Network, which aims to increase participation and advancement of women in the academic STEM fields. Our team of researchers focused on comparing the two STEM disciplines of chemistry and physics, because both are experimental sciences that offer many opportunities for undergraduate students to participate in research projects. The one-on-one approach where students are integrated into ongoing experimental, theoretical, or computational research projects is similar in these two STEM fields. Ten years of data about undergraduate research in the fields of physics and chemistry from four different PUI’s across the United States were collected with the intention that analysis of these current data would provide insight into what factors impact the productivity of undergraduate research students.

The universities involved in this study are private and, with one exception, Catholic institutions, each enrolling between 1600 and 4000 undergraduates in their liberal arts colleges. Undergraduates are mostly traditional students, enrolling in college right after their high school graduation. Bachelor of Science degrees are the highest degrees offered in both Physics and Chemistry at each institution, excepting one university, which has a Master’s program in Physics. All four institutions have high faculty teaching loads along with the expectation that tenure-track and tenured faculty engage in research and involve undergraduates in their research efforts.

At each institution, undergraduate research plays an important role in the college experience and is made available to interested students both during the academic year and the summer. During the investigated timeframe, between 30% and nearly 100% of graduating chemistry or physics students at each of these institutions participated in undergraduate research in physics and chemistry. Depending on the institution, students obtain a faculty research advisor by either directly interacting with a faculty member whose research interests the student or by submitting an application to the STEM department or a committee, who assigns the student to a faculty mentor. Effort is made by every faculty member involved in research to accommodate interested students; at some institutions, junior and senior students receive preference if there are not enough positions. Depending on the institution, student research may be taken for credit—for example, in the form of an upper-level research methods course—but physics and chemistry students are *not* required to complete a research project to graduate, as is the case at some institutions. Typically, the research products in this study (presentations, posters, papers) require students to work on their research project for more than one semester.

### Data collection

This study examines undergraduate research products generated over a period of 10 years (2004–2013) at each of the four institutions. During the time frame, 59 faculty research advisors worked with a total of 548 research students (Table 1). Most of the research students were chemistry or physics majors, but some students were from other departments. All student researchers were included regardless of their academic major, because the research domain of the advisor is the most indicative variable of the type of research conducted. The following data were collected for each research product: gender of student, type of research product (poster presentation, oral presentation, institutional article, peer-reviewed article), type of venue (on-campus, local, regional or national venue), gender of advisor, and academic rank of advisor. In the dataset, each student was assigned an individual code and each of his or her research products were identified separately. For example, a peer-reviewed paper with three student co-authors would count as a research product for each of the three students. Any or all of those students may also have poster and/or oral presentations.

**Table 1:** Research participants and research products for 2004–2013 “Students” accounts for each individual reported as an author on a research product, “Advisors” accounts for each faculty member listed as an author. “Student Research Products” accounts for authorship credit of a research product (institutional paper, oral presentation, poster presentation, and peer-reviewed article). Gender distribution of faculty advisors also shown.

	Student Authors		Student Research products		Advisors	
	N	Percentage	N	Percentage	N	Percentage
**Chemistry**						
Female	213	55%	556	54%	10	24%
Male	175	45%	469	46%	31	76%
**Physics**						
Female	53	33%	171	36%	5	28%
Male	107	67%	301	64%	13	72%
**Total**	548		1497		59	

The data were collected via a census of the faculty, through records maintained by departments and institutions, and a search of online publication databases. Publications and presentations with undergraduates are part of faculty evaluation, and as such, appear on Curricula Vitae (CVs), in institutional archives of scholarly events and faculty publications, and in departmental or honor’s program databases of student research participants and their presentations. Each institution maintains records of undergraduate research products in different ways. By collecting and comparing the data from multiple sources, as complete a list as possible was generated. As each institution is small, this was a manageable task. Any unclear data points were clarified with faculty advisors. Data from faculty who were not active in research or whose authorship of research products was not available are not part of this study. Additionally, the data collection process did not control for student intention to pursue professional or graduate school or a job after college graduation. The data collection process did not account for a student’s skill level or for the interplay of student and faculty self-efficacy and attitudes that may impact research productivity.

Some of the research products in this study (for example, presentations at regional or national meetings) require travel. Travel opportunities are made available to research students with no regard for gender. There are no predetermined (or even encouraged) gender quotas for travel set by the departments or the universities. The number of students traveling depends on available funding, quality of the students’ research projects, and, in some cases, on the availability of accompanying faculty members.

### Data analysis

For this study, the data were combined from all participating institutions and were split by teaching discipline of the research mentor (chemistry or physics). The data were not subdivided further because the female population linked to physics for both professors and students at the four institutions was small and could potentially identify individual faculty and students. The data were analyzed with descriptive statistics followed by a series of Generalized Linear Mixed Models (GLMM) with bootstrapping. While the descriptive statistics give a basic understanding of the sample data, there are two reasons to look for a more appropriate statistical model to understand patterns that influence the distribution of undergraduate research products. One reason is to statistically identify the significant variables affecting the research products of undergraduates in PUIs and the other is to solve the issue of the small sample size in the current dataset. As described below in detail, Generalized Linear Mixed Models were used to identify the variables that impact the undergraduate research products and bootstrapping was used to overcome the small sample size. The results of the descriptive statistics will be presented first followed by an explanation of the GLMM methods and the results of that analysis.

## Results

### Overview of collected data

#### Research product patterns by student gender

From 2004 to 2013, 548 undergraduate students at the four PUI’s authored research 1497 products in chemistry and physics. Table 1 shows that gender distribution of student authors (individuals reported as authors on any research product) and research products (as explained above, one individual can have multiple research products) follow the same pattern within each discipline. In chemistry, 55% of the students who authored a research product are females and 54% of the research products have a female author. In physics, 33% of the student authors are females and 36% of the research products have a female author. The 3% difference between authors and research products in physics is explained by outliers in the distribution of research products (see S2 Fig in the SI (supplementary information)). Because the pool of female students conducting research in physics is the smallest in our sample, outliers carry a stronger weight and thus have a stronger effect in the calculation of the percentages. Overall, female students author a research product at the same relative proportion as male students, with a median of two research products per student. (See S1 Table)

As a point of reference, national statistics of graduates for 2011–12 at postsecondary institutions, with no distinction between PUIs and research-intensive universities for the aggregated data, indicate that 48% of total graduates in chemistry are female and 19% of total graduates in physics are female [51]. While these percentages do not correspond to research involvement, they provide a national context for this study in which only research activity at PUIs is examined. The percentage of female undergraduate researchers involved in undergraduate research observed here (55% of the chemistry researchers and 33% of the physics researchers are female) is higher than the national statistics for total graduates [35].

#### Distribution of research products

The 548 undergraduate students involved in research authored 1497 research products, consisting of poster presentations, oral presentations, and peer-reviewed publications (Table 2).

**Table 2:** Number of different types of research products and the venue of the research product by each gender in each discipline. Percentages of research products by female students are shown in parentheses. In general, these percentages are similar to the overall distribution of student authors and student research products shown in Table 1. For a bar graph of the percentages, see S3 and S4 Figs.

	Type of Research Product				Venue	
	Peer reviewed article	Institutional paper	Oral presentation	Poster presentation	On-campus or local	Regional or national
**Chemistry**						
Female	70	43	97	346	183	260
	(59%)	(75%)	(55%)	(51%)	(55%)	(50%)
Male	49	14	78	328	148	258
**Physics**						
Female	32	8	51	80	36	9
	(31%)	(40%)	(34%)	(41%)	(33%)	(40%)
Male	72	12	100	117	73	143

In undergraduate research at the institutions involved in this study, poster presentations are the most common research products in both disciplines (chemistry: 674 posters; physics: 197 posters, see Table 2) for both male and female students. A poster in undergraduate research features, in many cases, several authors because each research project may involve modular sub-projects which are the work of different undergraduates. Posters presented at institutional (on-campus), local, regional or national venues were all included in this study. An institutional poster is, in most cases, presented at on-campus events aimed at showcasing the research projects of students at 4-year colleges. Poster presentations on the national level are often the result of efforts made by professional associations, such as the American Chemical Society, to devote sessions at national meetings exclusively to presentations of posters by undergraduates and the rise of national conferences dedicated exclusively to undergraduate research presentations (such as the National Conference on Undergraduate Research which started in 1987 as part of the Council for Undergraduate Research). Overall, the high number of posters for undergraduate researchers is unsurprising as this is often an initial entry point into scientific presentation.

The lower number of oral presentations, 175 in chemistry and 151 in physics, is an effect of one author per oral presentation, as opposed to the likelihood for more than one author per poster, and to fewer opportunities for oral presentations at on-campus events due to scheduling and time availability of the academic community at 4-year colleges. A similar observation applies for oral presentations at regional or national venues, where opportunities for oral presentations by undergraduate students are less common.

The most prestigious research product for undergraduate research is arguably the peer-reviewed publication. In our database we found 119 peer-reviewed papers in chemistry and 104 in physics. Undergraduates conducting research in physics author a peer-review article at a greater percentage than in chemistry, which may be an effect of the publishing culture in each discipline. Female students conducting research with a physics faculty author a paper at a lower proportion than male students. For peer-reviewed publications, females in chemistry show a slightly greater percentage of papers while in physics females show a lower percentage of papers. (See Table 2.)

#### Research product patterns by venue

The distribution of research products for both genders in each discipline were further investigated with respect to venue. From the 1197 total poster and oral presentation entries recorded in the database, 63% correspond to regional or national presentations. Table 2 shows that the percentages of research products authored by venue in both disciplines are quite similar to their corresponding overall patterns seen in Table 1.

A number of factors may influence whether undergraduate students present their research at the local, regional or national level. Availability of local and regional venues to present undergraduate research vary depending on the location of the institution (city and state), and the existence of local and regional programs that support presentations by undergraduate students. In some cases, chapters of professional associations organize regional conferences where students present their research; federal or state funding may support state-wide conferences targeted at undergraduates, while private or philanthropic initiatives may also provide opportunities for research presentation by undergraduates. Four-year PUI’s provide funding, limited in some instances, for students and faculty to attend regional or national meetings. Initiatives by organizations like the American Chemical Society, which has dedicated sessions at national meetings for undergraduate posters and oral presentations (while still welcoming undergraduate presentations in disciplinary sessions) as well as special seminars and programming for undergraduate attendees, provide opportunities for students to disseminate their research products at national venues.

#### Undergraduate student and research mentor gender match

To investigate whether the completion of research products is influenced by the gender of the research mentor, the data were inspected by analyzing the distribution of research products clustered by gender match between student and research mentor (Table 3). *Gender match* is defined as the student and faculty member having the same self-identified gender where *no match* indicates different self-identified genders of the student and the faculty member. Overall, the percentages of gender match for different research products are almost equivalent in both disciplines except for peer- reviewed articles in physics (See Table 3 below and S5 Fig). This exceptional behavior in physics could be due to its relatively small data size, and additional statistical analysis to answer the question of significance of gender match will be presented below.

**Table 3:** Number of different research products by gender match in each discipline.

	Peer reviewed articles	Institutional papers	Oral presentations	Poster presentations
**Chemistry**				
gender match	50	19	81	361
no match	69	38	94	312
**Physics**				
gender match	72	15	80	103
no match	32	5	71	94

### Statistical analysis using Generalized Linear Mixed Models with hierarchical bootstrapping

In evaluating possible approaches to a more detailed statistical analysis of this dataset, a simple linear regression model is not an appropriate choice. With linear regression, it is assumed that the observations are independent from one another and fall into one common group that holds common correlation characteristics. This feature is called fixed effects, which are constants and are estimated from the data. In contrast, when the observations are not independent and the measurements of the response variable are grouped according to some structure (such as colleges, classes, countries, experimental units, etc.) or a hierarchy, there is a possible correlation among the measurements in each group (observations within a group are correlated), violating the independence of the observations. This subgrouping of data that is due to the hierarchy adds random effects. Random effects are identified by the structure of hierarchy. For example, if the observations are subgrouped by classes, then class will be a possible random effect variable. When a significant hierarchical structure is present, it requires incorporating both the random effects that came from the hierarchical structure of the data and also the fixed effects from those independent observations under each level of the hierarchy together in the model. “Mixed effects models” or simply “mixed models” are used to handle both of these effects in regression models [52].

The dataset under study consists of data that form a hierarchy from each PUI to each faculty member to each student. There are multiple students under each faculty member, and there are multiple faculty members considered under each PUI. Within each subgroup, possible correlations could occur among the observations within each of these subgroups. For example, it is possible that a group of students who work under one faculty member work similarly due to the influence of that faculty member and also work differently from a group of students who work under a different faculty member. This hierarchy and the grouping structure affect the independency of the observations, and it was necessary to capture the random effects into the model, leading to the choice of Mixed Models (MM).

When modeling this dataset with MM, there are two objectives to achieve. One objective is to model the number of research products as the response variable. The total number of research products is a count (0, 1, 2, 3,…) and also non-negative. For a variable that allows non-negative counts without an upper limit, a Poisson distribution is the best choice [53] (note that a Binomial random variable has an upper limit: the values range from 0 up to an upper limit of n). Therefore, when modeling the number of research products, the response variable follows a Poisson distribution. This is different from linear regression. In linear regression models, the response variable follows a normal distribution. For a continuous response variable that follows a normal distribution, a linear regression model is the best choice. Therefore, as the response variable in this case follows a Poisson distribution, a more general version of linear regression models, known as Generalized Linear Models (GLM), is appropriate. GLM extends the well-known linear regression models when the response variable does not necessarily need to be normal. Since the response variable follows a Poisson distribution, it is necessary to use a GLM with a Poisson response variable. This is also called “Poisson regression”. GLM, similar to linear regression, models only fixed effects. Following the discussion about the need of a mixed model for this data, the Poisson regression model is required to incorporate mixed effects into the model. Note that GLMM is a combination of GLM and MM that combines both random and fixed effects. Therefore, the number of research products was modeled using mixed effects Poisson regression. Furthermore, the mixed effect Poisson regression models that are presented in Tables 4 and 5 were checked for overdispersion. Poisson random variables assume the mean and the variance are equal. When the variance is greater than the mean, an overdispersion occurs [54]. The mixed effect Poisson regression models that are presented in Tables 4 and 5 resulted in estimated dispersion parameters (ratio of the variance to the mean) that are very close to 1 (chemistry: 1.101435 and physics: 1.100499), showing no overdispersion.

**Table 4:** Bootstrap results of mixed effect Poisson regression for chemistry data on 1000 bootstrap repetitions. The response variable of total number of research products was modeled using student gender, faculty rank, venue, and gender match as input variables with faculty identifier as a random effect as explained in the GLMM Approach section. Note that faculty rank and venue are significant.

Model: total number of research products = student gender + faculty rank + venue + gender match. Random effect: faculty				
	Estimate	standard error	95% confidence interval	
Intercept	1.134	0.111	0.859	1.561
student gender = female	-0.174	0.036	-0.290	0.149
faculty rank = associate professor	-**0.383**	0.042	-**0.608**	-**0.023**
faculty rank = full professor	-**0.692**	0.155	-**1.190**	-**0.085**
venue = regional or national	**0.161**	0.038	**0.004**	**0.280**
student—faculty gender match	0.202	0.036	-0.147	0.321
standard deviation of random effect	0.555	NA	0.371	0.708

**Table 5:** Bootstrap results of mixed effect Poisson regression for physics data on 1000 bootstrap repetitions. The response variable of total number of research products was modeled using student gender, faculty rank, venue, and gender match as input variables with faculty identifier as a random effect as explained in the GLMM Approach section. Note that only the gender match is significant.

Model: total number of research products = student gender + faculty rank + venue + gender match. Random effect: faculty				
	Estimate	standard error	95% confidence interval	
Intercept	0.800	0.128	0.426	1.294
student gender = female	0.092	0.073	-0.347	0.330
faculty rank = associate professor	0.036	0.071	-0.723	0.302
faculty rank = full professor	0.329	0.195	-0.417	1.108
venue = regional or national	0.208	0.076	-0.001	0.415
student—faculty gender match	**0.477**	0.074	**0.025**	**0.723**
standard deviation of random effect	0.350	NA	0.143	0.565

The second objective is to model the likeliness of a particular type of research product (article, poster, oral presentation). For this objective, the response variable is binary (1 or 0) under each type of research product. For example, one of the models was to check how likely it is for a student to produce a poster presentation based on the characteristics under consideration (gender, faculty advisor, etc). With a binary response variable, the best candidate is logistic regression [52]. Due to the hierarchical structure in the data, a mixed effects logistic regression model was used.

For this data, there are eight (8) different models. To analyze the total number of research products within each discipline (chemistry and physics), two (2) mixed effect Poisson regression models are used. To evaluate the three types of research products (article, poster, oral presentation) in both disciplines (chemistry and physics), there are six (6) mixed effect logistic regression models. Different combinations of fixed effect variables and random effect variables were tested in both mixed effect Poisson regression and mixed effect Logistic regression.

One important finding was that the models with faculty identifier as the random effect produced the best results for the model evaluation criteria when compared to the models with PUI as a random effect variable and to the models with both faculty identifier and PUI together as random effects. The best models were identified by comparing the AIC (Akaike Information Criterion) and BIC (Bayesian Information Criterion) of the models. Smaller values of AIC and BIC indicate better models. The GLMM with faculty identifier produced the smallest values for AIC and BIC when comparing to the models with PUI as a random effect variable and to the models with both faculty identifier and PUI as random effect variables. As a result, GLMMs with faculty identifier as a random effect were determined to be the best models and are used in the remainder of the paper.

The datasets used for this study are from a total of 388 students in chemistry and 160 students in physics. In total, those 548 students have produced 1497 undergraduate research products. This is a relatively small sample. To overcome the effect of the small sample size, bootstrapping was applied to parameter estimation. Bootstrapping is a commonly used resampling technique. When the sample of data used in a study is not sufficiently large or when it is difficult to make multiple samples from the population, bootstrapping helps by creating samples from the existing sample [55]. In this case, the existing sample serves as the “population” to the multiple bootstrap samples. These multiple bootstrap samples aid in understanding the characteristics of the different statistics presented. For example, these multiple bootstrap samples can be used to create confidence intervals of the parameter estimates in different GLMMs. When using bootstrapping, it is important to preserve the original characteristic of the sample data. Since our data form a hierarchy, it was imperative to maintain the same hierarchy when resampling from the dataset. To maintain the hierarchy, hierarchical bootstrapping was used [53]. The bootstrap confidence intervals based on 1000 bootstrap samples for each parameter estimate are presented in Tables 4, 5, 6 and 7. All the models were implemented using the statistical software ‘R’.

**Table 6:** Bootstrap results of mixed effect logistic regression for chemistry data on 1000 bootstrap repetitions. The response variable of log odds of a particular type of research product was modeled using student gender and gender match as input variables with faculty identifier as a random effect as explained in the GLMM Approach section. Note that neither student gender nor gender match is significant.

Model 1: log odds of an oral presentation = student gender + gender match Random effect: Faculty				
	Estimate	standard error	95% confidence interval	
Intercept	-1.614	0.214	-2.343	-1.156
student gender = female	-0.036	0.192	-0.726	0.588
student—faculty gender match	0.126	0.192	-0.467	0.803
standard deviation of random effect	0.766	NA	0.690	1.673
Model 2: log odds of a peer reviewed article = student gender + gender match Random effect: Faculty				
	Estimate	standard error	95% confidence interval	
Intercept	-2.415	0.281	-3.580	-1.974
student gender = female	0.070	0.260	-0.776	0.741
student—faculty gender match	0.426	0.260	-0.276	1.128
standard deviation of random effect	0.981	NA	0.731	1.975
Model 3: log odds of a poster presentation = student gender + gender match Random effect: Faculty				
	Estimate	standard error	95% confidence interval	
Intercept	0.710	0.213	0.162	1.162
student gender = female	-0.138	0.168	-0.715	0.456
student—faculty gender match	-0.351	0.167	-0.942	0.191
standard deviation of random effect	0.923	NA	0.752	1.764

**Table 7:** Bootstrap results of mixed effect logistic regression for physics data on 1000 bootstrap repetitions. The response variable of log odds of a particular type of research product was modeled using student gender and gender match as input variables with faculty identifier as a random effect as explained in the GLMM Approach section. Note that neither student gender nor gender match is significant.

Model 1: log odds of an oral presentation = student gender + gender match Random effect: Faculty				
	Estimate	standard error	95% confidence interval	
Intercept	-0.804	0.227	-1.596	-0.924
student gender = female	-0.253	0.287	-0.685	0.397
student—faculty gender match	0.243	0.283	-0.383	0.723
standard deviation of random effect	0.662	NA	0.152	0.693
Model 2: log odds of a peer reviewed article = student gender + gender match Random effect: Faculty				
	Estimate	standard error	95% confidence interval	
Intercept	-2.670	0.847	-5.667	-1.724
student gender = female	0.375	0.907	-0.595	1.317
student—faculty gender match	-0.299	0.907	-1.287	0.691
standard deviation of random effect	2.478	NA	0.987	3.961
Model 3: log odds of a poster presentation = student gender + gender match Random effect: Faculty				
	Estimate	standard error	95% confidence interval	
Intercept	-0.661	0.439	-2.376	-0.749
student gender = female	0.072	0.309	-0.500	0.493
student—faculty gender match	-0.085	0.309	-0.587	0.357
standard deviation of random effect	1.604	NA	0.290	2.013

The main purpose of this study is to identify the factors that influence the completion of student research products at PUIs. To contextualize this study and harmonize all eight mixed models, GLMMs with both significant and non-significant variables will be shown. This will highlight the relationship of the predictor variables with the response variable, if any, and identify the influential and non-influential factors for the corresponding response variables, leading the statistical analysis towards “detection” rather than “prediction.” Therefore, for the task of detecting relationship, model statistics such as explained deviation (pseudo R^2^) are not relevant because the nature of the relationship of the variables does not change depending on the value of the R^2^ [56]. However, statistics that describe the effects of random and fixed effects in the model will be presented. The measure of the variability of the random effects in the model, parameter estimates corresponding to the predictor variables, and bootstrap confidence intervals will also be presented. The measure of the variability of the random effects describes the amount of variation added by the random effect variable (the variable that subgroups the dataset) to the model. A useful random effect variable is expected to a have a significant non-zero variability added to the model. The bootstrap confidence intervals will be used as a way of deciding the significance of each parameter and hence the usefulness of the corresponding predictor variable in the model.

Tables 4 and 5 show the bootstrap results of mixed effect Poisson regression for the chemistry and physics data sets with the total number of research products of each student as the response variable. The tables consist of natural logarithm values of the parameter estimates under the “Estimate” column in each table. Therefore, when interpreting the change in the total number of research products produced by a student, the exponential value of the corresponding parameter estimate will be taken. It is important to note that the confidence intervals for the standard deviation of the random effect (in this case Faculty) does not contain the value zero in all the eight mixed models presented here. This indicates that the variance (or the square of the standard deviation) of the random effect is significantly non-zero in all the mixed effect models, indicating that the use of the faculty as a random effect variable adds a significant variability to the mixed model. This shows that the research products of students of one faculty member have a similar pattern and are different from those of another faculty member.

For chemistry, the faculty ranks of associate professor and full professor as well as venue are significant (confidence intervals do not contain the value zero). The estimates suggest that as the faculty research mentor moves up in rank, the total number of student research products increases by less than 1 when all other factors are kept fixed. For example, when faculty rank increases from assistant professor to associate professor, the average number of research products will go up by 0.682 (or e^-0.383^), when all other factors remain the same. Similarly, when the faculty rank increases from assistant professor to full professor the average number of research products will go up by 0.501 (or e^-0.692^), when all other factors are kept fixed. In addition, by changing the venue from “on-campus or local” to “regional or national” the total number of research products will go up by 1.17 (or e^0.161^), when all the other factors remain the same. (Note that the parameter estimates should be converted to its corresponding exponential value). No significant student or faculty gender effect is observed.

For physics (Table 5), only the student-faculty gender match is significant (as the confidence interval does not contain zero). The parameter estimate (0.477) suggests that working with an advisor with the same gender as the student, will increase the average number of research products by 1.6 (or e^0.477^), when all other factor are fixed.

Tables 6 and 7 show six (6) different mixed effect logistic regression models of how likely each particular research product (oral presentations, poster presentation and peer-reviewed articles) is produced in each discipline (chemistry and physics). In both disciplines, it is clear that student gender and gender match are not influential in determining whether a student produces a particular type of research product (note that all confidence intervals contain the value zero).

The GLMM with hierarchical bootstrapping results show that student gender is not an important factor in determining the total number of undergraduate research products at the four PUIs studied here. (Tables 4 and 5). Further, gender does not play a role in producing each research product (Tables 6 and 7). The results indicate that faculty rank appears to be a minor factor in the generation of undergraduate research products for chemistry but not for physics. In physics, gender match is significant; the total number of research products increases by 1.6 when going from not matching genders to matching genders.

Previous work has shown that individual attributes of faculty members (training, professional contacts, research record, etc.) and structural factors (work environment, culture of department, public versus private institution, etc.) have more impact than faculty rank [57–59]. Given the similarity of departments and institutions of this data set, the differences that are seen in this data set based on faculty rank cannot be easily explained using the hypotheses suggested in the literature.

## Conclusions

This study of research products of undergraduates at four primarily undergraduate institutions, from 2004 to 2013 sets a benchmark for the assessment of undergraduate research efforts. Statistical models indicate that student gender or gender match of the student and faculty member had no impact on producing each type of research product (Tables 6 and 7) by undergraduate students at the PUIs in this study. While no significant effect due to participant gender was observed for type of research product, a number of factors did impact the total number of research products a student generated. In chemistry, students were likely to generate more research products if they presented at a regional or national venue (Table 4). This is perhaps a function of the location and culture of these institutions. More data from other PUIs is necessary to contextualize this finding. In physics, student-faculty pairs of the same gender generated more research products than mixed-gender pairs. While faculty rank appears to play a minor role in the chemistry data analyzed here (Table 4), the likely increase in the number of research products for a student is less than one if their advisor is below the rank of associate professor. Because all the models identified the faculty as the random effect with significant variance, it indicates that the group of students of one faculty member works differently from the group of students who work under a different faculty member.

It is clear that the culture of the institutions in this study promotes the participation of undergraduates in research experiences. There are, of course, a number of questions that remain to be addressed that require additional information from a broad range of institutions. A non-exhaustive list of these questions include the following: How do research product distributions compare to other PUIs and Tier 1 research universities? What role does faculty rank play at other PUIs and Tier 1 institutions? In what way do subdisciplines of chemistry and physics impact research product distribution? To what extent does dissemination of undergraduate research products influence students’ decisions on persistence in STEM (whether graduate school or job market) after college graduation? How does the research culture in light of gender compare between PUIs and research intensive universities?

To address these questions a broad, longitudinal study of research products at a wide variety of institutions and incorporating student surveys is necessary. We see an opportunity here for the academic community to provide a more accurate picture of the state of undergraduate research at the national level. Specifically, we encourage science departments at both the undergraduate and graduate level to collect and share their own data to provide a more robust view of the effectiveness of undergraduate research experiences at PUIs.

## Supporting information

S1 Fig Normalized distributions of the number of student products by gender in each discipline. The horizontal axis represents the number of products authored by an individual student. The vertical axis represents the relative frequency of the number of products.
(TIF)

S2 Fig Box plots of the number of research products for each gender in each discipline. The dark vertical line is the mean number of research products in the data set, and the two vertical lines beside the mean are 1^st^ and the 3^rd^ quartiles. The vertical line connected to the box with the dotted line is called the adjunct value—the maximum of the dataset after removing the outliers. Outliers appear as dots to the right of the vertical line.
(TIF)

S3 Fig Percentages of poster presentations, oral presentations, and peer-reviewed publications per discipline and per gender. For comparison, the leftmost two bars correspond to the percentage of student authors. Data that comprise these percentages are shown in Table 2 in the main manuscript.
(TIF)

S4 Fig Percentage of research products (including only oral and poster presentations) by location of venue per discipline and per gender. For comparison, the leftmost two bars correspond to the percentage of student authors.
(TIF)

S5 Fig Percentages of research product type per discipline based on student-research advisor gender match. (TIF)

S1 Table Summary statistics of research products per student. See S2 Fig for a boxplot of these data.
(DOCX)

S1 Text Outlier analysis of data and model selection criteria. (DOCX)
