# Supplementary figures and images for: Factors affecting the number and type of student research products for chemistry and physics students at primarily undergraduate institutions: A case study
Source: PLoS One. 2018 Apr 26;13(4):e0196338. doi: 10.1371/journal.pone.0196338 (PMC5919462; doi:10.1371/journal.pone.0196338)

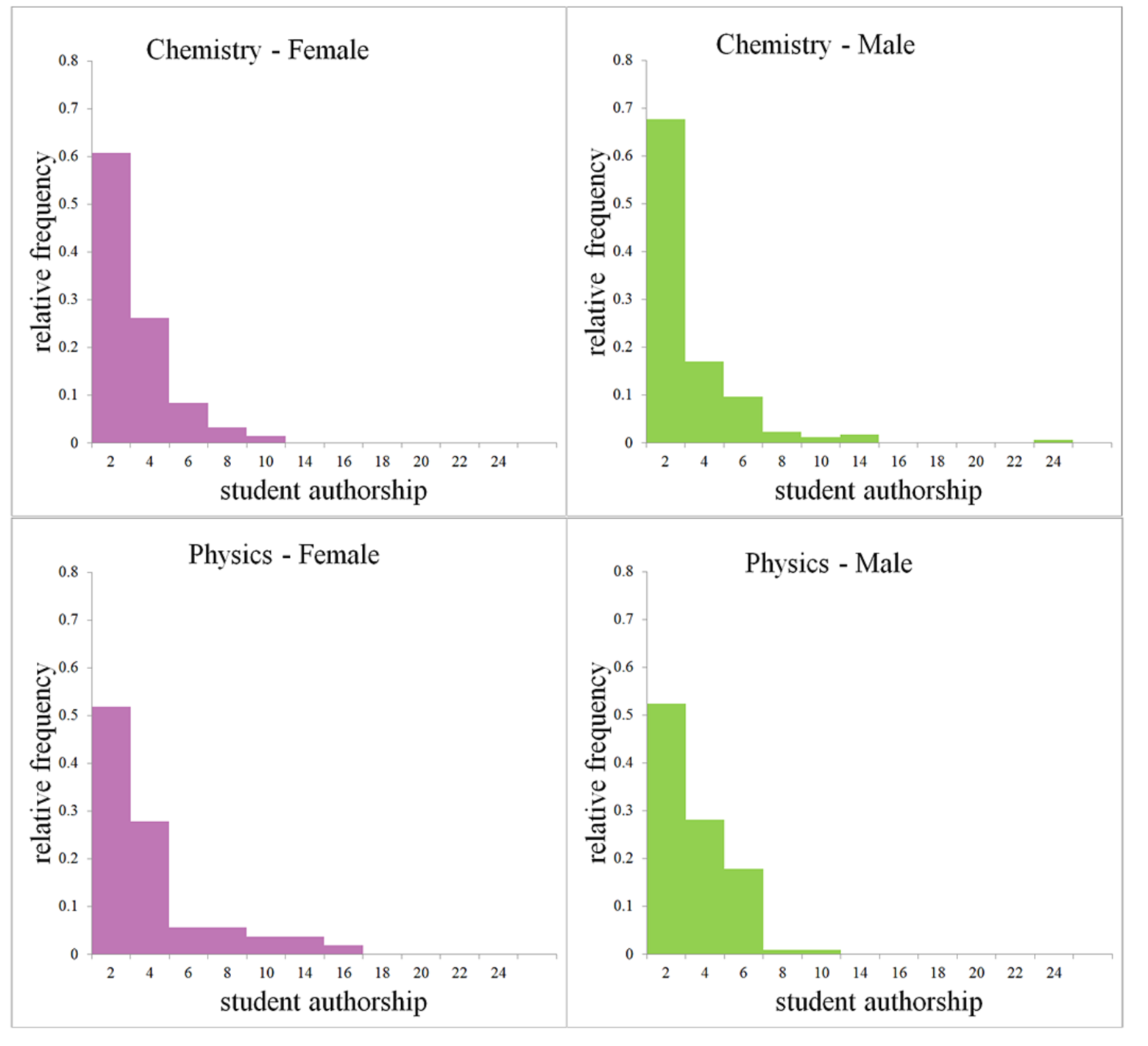

Supplement: S1 Fig — The horizontal axis represents the number of products authored by an individual student. The vertical axis represents the relative frequency of the number of products. (TIF) [file pone.0196338.s001.tif]

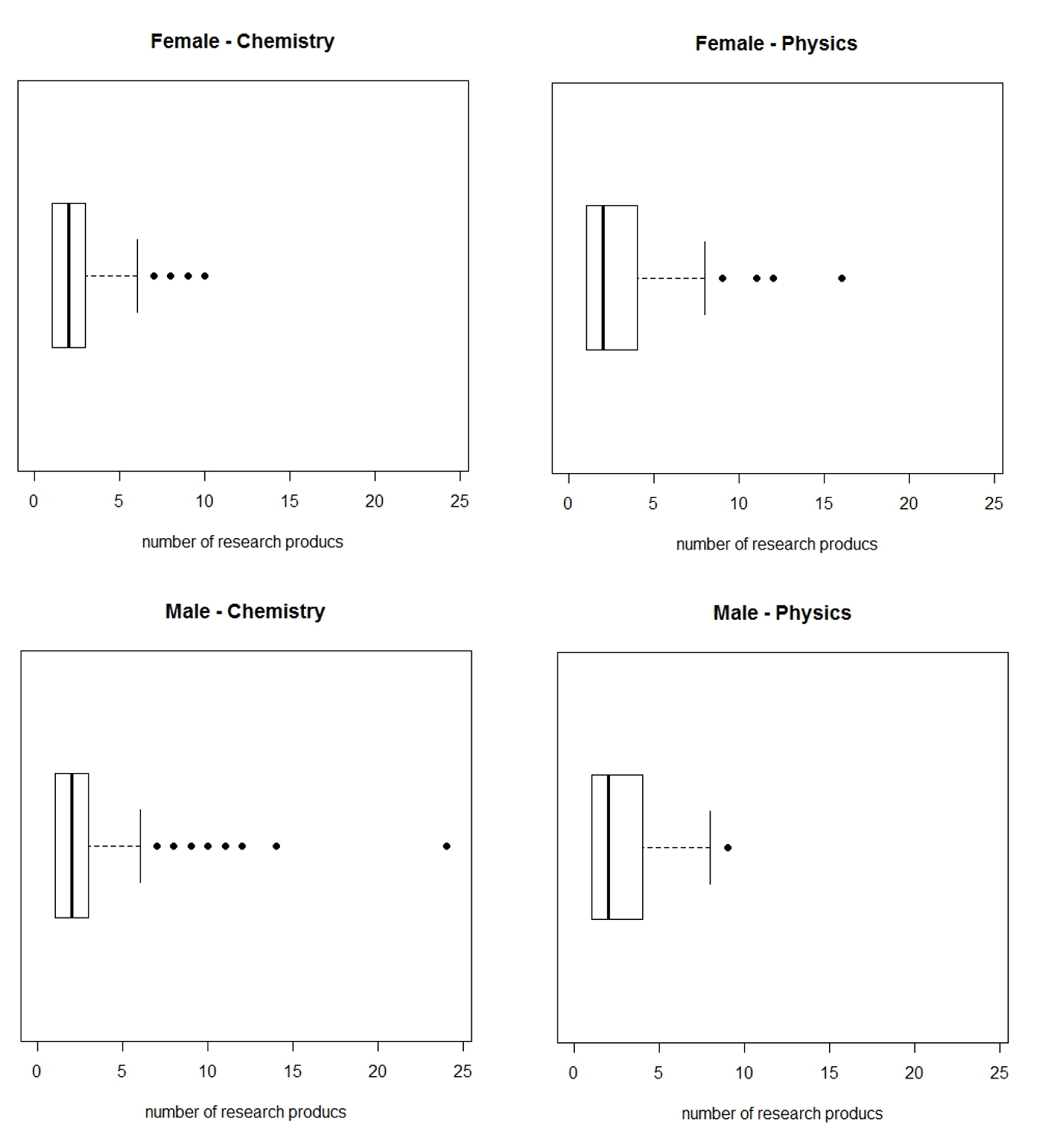

Supplement: S2 Fig — The dark vertical line is the mean number of research products in the data set, and the two vertical lines beside the mean are 1st and the 3rd quartiles. The vertical line connected to the box with the dotted line is called the adjunct value—the maximum of the dataset after removing the outliers. Outliers appear as dots to the right of the vertical line. (TIF) [file pone.0196338.s002.tif]

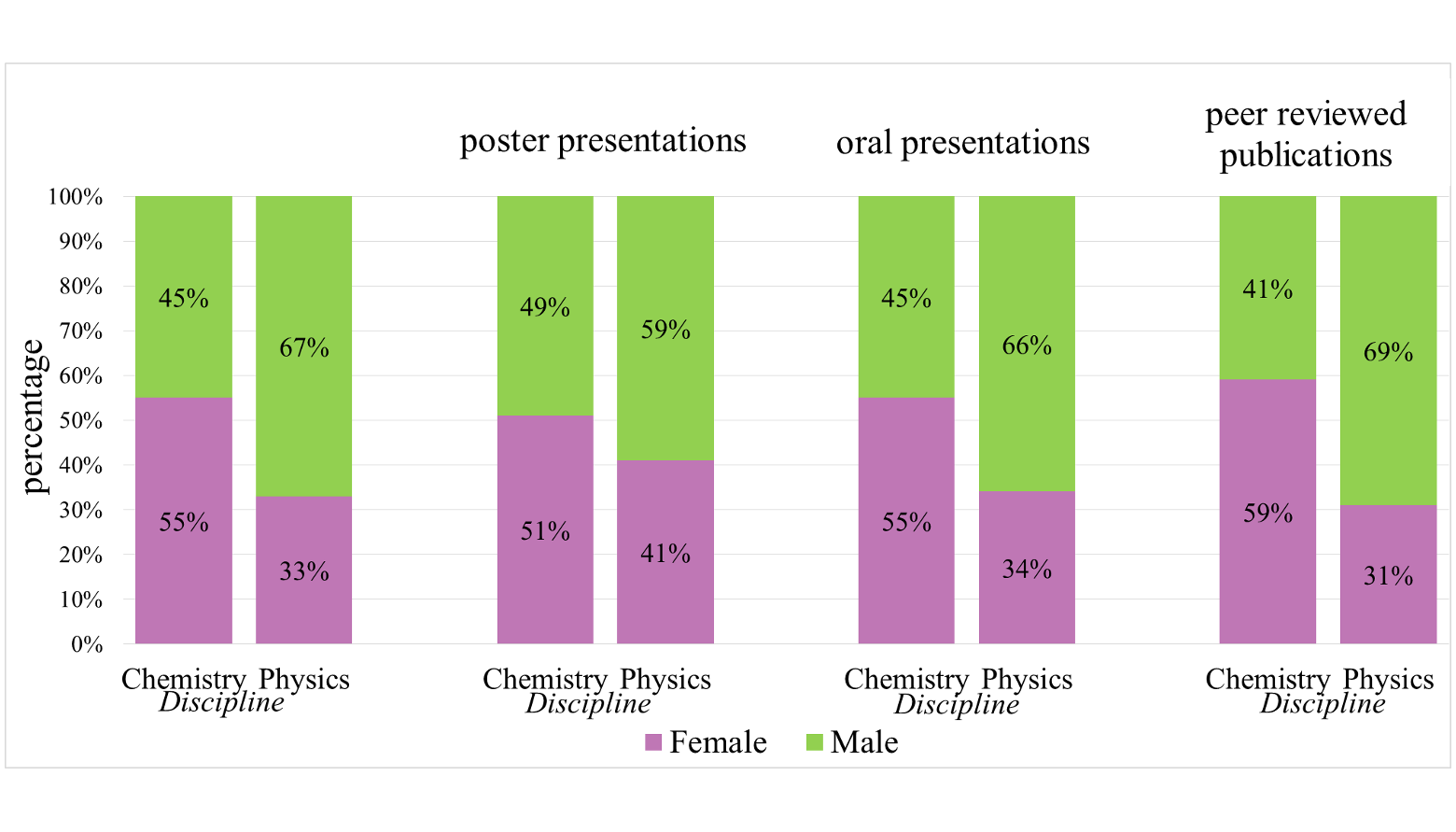

Supplement: S3 Fig — For comparison, the leftmost two bars correspond to the percentage of student authors. Data that comprise these percentages are shown in Table 2 in the main manuscript. (TIF) [file pone.0196338.s003.tif]

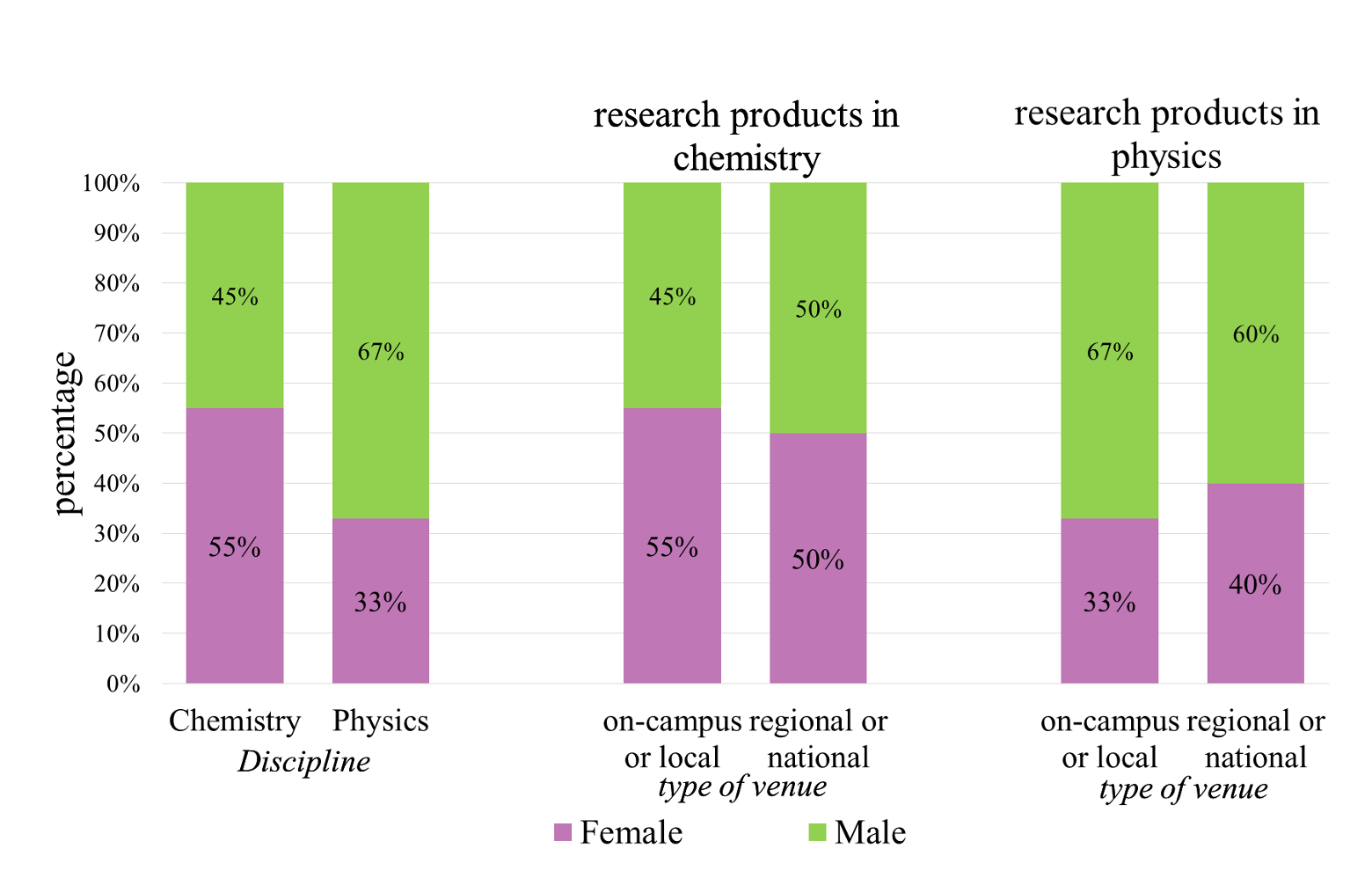

Supplement: S4 Fig — For comparison, the leftmost two bars correspond to the percentage of student authors. (TIF) [file pone.0196338.s004.tif]

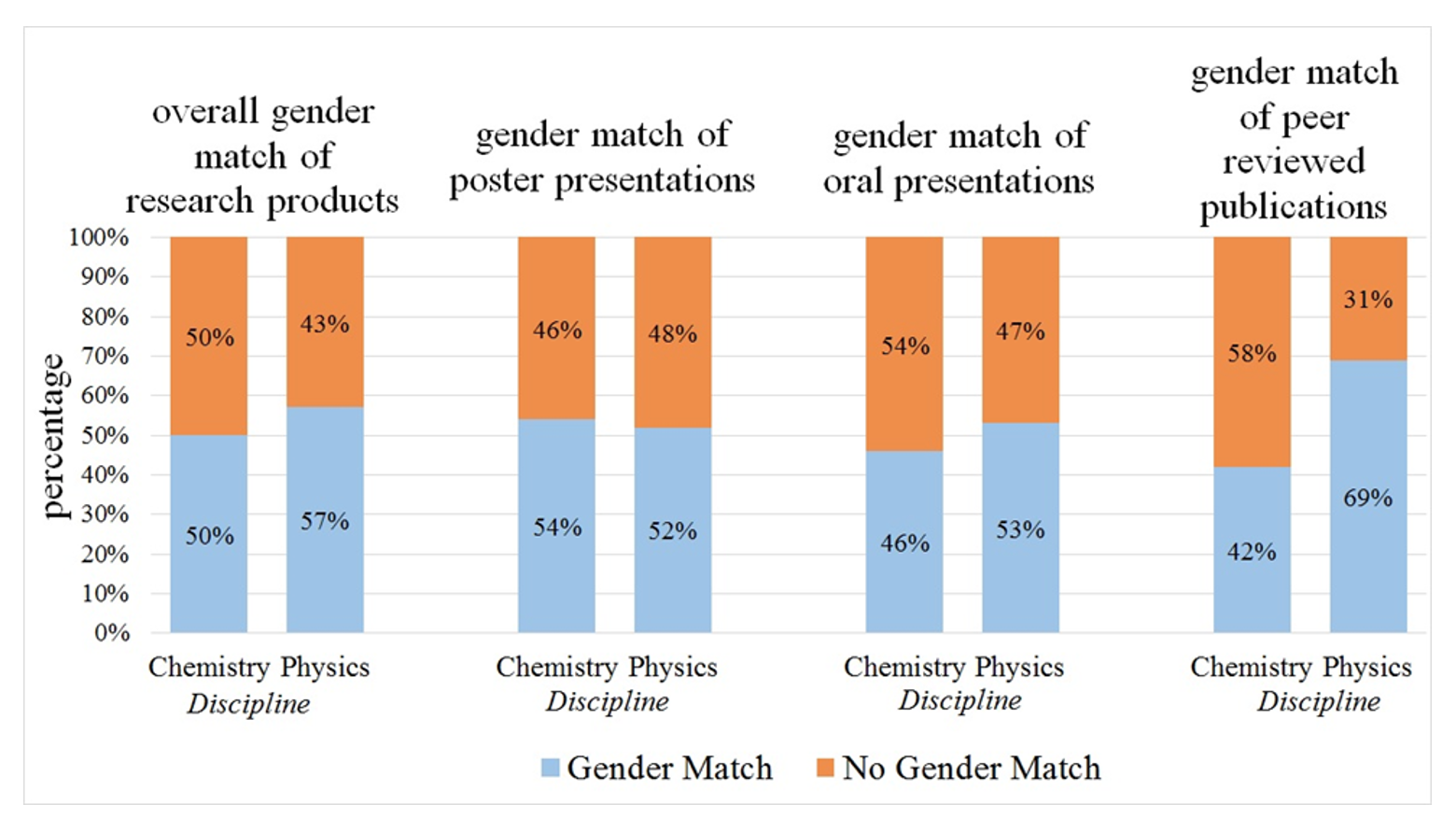

Supplement: S5 Fig — (TIF) [file pone.0196338.s005.tif]
