# Supplementary material for: Factors affecting the number and type of student research products for chemistry and physics students at primarily undergraduate institutions: A case study
Source: PLoS One. 2018 Apr 26;13(4):e0196338. doi: 10.1371/journal.pone.0196338 (PMC5919462; doi:10.1371/journal.pone.0196338)
Supplement: S1 Table — See S2 Fig for a boxplot of these data. (DOCX) [file pone.0196338.s006.docx]

|  |  | **Number of Student Research Products** | | | |
| --- | --- | --- | --- | --- | --- |
|  |  | Median | Mmean | 1^st^ quartile quartile | 3^rd^ quartile |
| **Chemistry** |  |  |  |  |  |
| Female |  | 2.0 | 2.6 | 1.0 | 3.0 |
| Male |  | 2.0 | 2.7 | 1.0 | 3.0 |
| **Physics** |  |  |  |  |  |
| Female |  | 2.0 | 3.2 | 1.0 | 4.0 |
| Male |  | 2.0 | 2.8 | 1.0 | 4.0 |
|  |  |  |  |  |  |
