# Supplementary material for: Factors affecting the number and type of student research products for chemistry and physics students at primarily undergraduate institutions: A case study
Source: PLoS One. 2018 Apr 26;13(4):e0196338. doi: 10.1371/journal.pone.0196338 (PMC5919462; doi:10.1371/journal.pone.0196338)
Supplement: S1 Text — (DOCX) [file pone.0196338.s007.docx]

**Factors affecting the number and type of student research products for chemistry and physics students at primarily undergraduate institutions: a case study**

Supplemental Information

**S1 Fig**. **Normalized distributions of the number of student products by gender in each discipline.** The horizontal axis represents the number of products authored by an individual student. The vertical axis represents the relative frequency of the number of products.

**Outlier Analysis of Data**

For both chemistry and physics, outlier analyses were performed and found few outliers. For each gender in each discipline, the upper and lower limits of the number of research products were calculated. Any data point outside the lower and upper limits are considered as outliers. For chemistry, the number of research products for both male and female students had a lower limit of -2 and an upper limit of 6. For physics, the number of research products for both male and female students had a lower limit of -3.5 and an upper limit of 8.8. No data points were below the respective lower limits, as the number of research products is a non-negative value. Boxplots in Figure S2 show the outliers (shown as filled circles on the right of each plot) in each dataset. The outliers represent a very small subpopulation in the database (12 in chemistry: 4 females and 8 males; and 5 in physics: 4 females and 1 male) and thus further statistical analysis on this subpopulation was not possible~~.~~

**S2 Fig. Box plots of the number of research products for each gender in each discipline.** The dark vertical line is the mean number of research products in the data set, and the two vertical lines beside the mean are 1^st^ and the 3^rd^ quartiles. The vertical line connected to the box with the dotted line is called the adjunct value – the maximum of the dataset after removing the outliers. Outliers appear as dots to the right of the vertical line.

**S1 Table. Summary statistics of research products per student**. See S2 Fig for a boxplot of these data.

**S3 Fig.** Percentages of poster presentations, oral presentations, and peer-reviewed publications per discipline and per gender. For comparison, the leftmost two bars correspond to the percentage of student authors. Data that comprise these percentages are shown in Table 2 in the main manuscript.

**S4 Fig.** Percentage of research products (including only oral and poster presentations) by location of venue per discipline and per gender. For comparison, the leftmost two bars correspond to the percentage of student authors.

**S5 Fig.** Percentages of research product type per discipline based on student-research advisor gender match.

**Model Selection Criteria**

The best models were identified based on Akaike Information Criterion (AIC), Bayesian Information Criterion (BIC) [52], and residual deviance (also known as deviance). AIC and BIC are two of the commonly used model selection criteria. Residual deviance is used in generalized linear models and is equivalent to the residual sum of squares in linear regression [59]. In AIC, BIC and also residual deviance, smaller values indicate that the model is better. “Inter Class Correlation” (ICC) was used to identify the correlation within a subgroup [59]. Higher ICC indicates that observations within are group a highly correlated, which further support the need of a mixed effects model [59]. In generalized linear models (and therefore in GLMM), “explained deviation”, which is also known as “pseudo R^2^”, is used in place of coefficient of determination (R^2^) [59]. Explained deviation has the similar interpretation as of the coefficient of determination in linear regression models. The GLMM with faculty identifier produced the smallest values for AIC, BIC, and residual deviance and produced highest values for ICC, indicating a better model with observations that are highly correlated under each faculty member.

Reference numbers refer to the main manuscript.
